# Targeted Silencing of Elongation Factor 2 Kinase Suppresses Growth and Sensitizes Tumors to Doxorubicin in an Orthotopic Model of Breast Cancer

**DOI:** 10.1371/journal.pone.0041171

**Published:** 2012-07-20

**Authors:** Ibrahim Tekedereli, S. Neslihan Alpay, Clint D. J. Tavares, Zehra E. Cobanoglu, Tamer S. Kaoud, Ibrahim Sahin, Anil K. Sood, Gabriel Lopez-Berestein, Kevin N. Dalby, Bulent Ozpolat

**Affiliations:** 1 Department of Experimental Therapeutics, University of Texas M. D. Anderson Cancer Center, Houston, Texas, United States of America; 2 Graduate Program in Cell and Molecular Biology, The University of Texas, Austin, Texas, United States of America; 3 Division of Medicinal Chemistry, College of Pharmacy, The University of Texas, Austin, Texas, United States of America; 4 Department of Gynecologic Oncology, University of Texas M. D. Anderson Cancer Center, Houston, Texas, United States of America; 5 Department of Cancer Biology, University of Texas M. D. Anderson Cancer Center, Houston, Texas, United States of America; 6 Center for RNAi and Non-Coding RNA, University of Texas M. D. Anderson Cancer Center, Houston, Texas, United States of America; Wayne State University School of Medicine, United States of America

## Abstract

Eukaryotic elongation factor 2 kinase (eEF-2K), through its phosphorylation of elongation factor 2 (eEF2), provides a mechanism by which cells can control the rate of the elongation phase of protein synthesis. The activity of eEF-2K is increased in rapidly proliferating malignant cells, is inhibited during mitosis, and may contribute to the promotion of autophagy in response to anti-cancer therapies. The purpose of this study was to examine the therapeutic potential of targeting eEF-2K in breast cancer tumors. Through the systemic administration of liposomal eEF-2K siRNA (twice a week, *i.v.* 150 µg/kg), the expression of eEF-2K was down-regulated *in vivo* in an orthotopic xenograft mouse model of a highly aggressive triple negative MDA-MB-231 tumor. This targeting resulted in a substantial decrease in eEF2 phosphorylation in the tumors, and led to the inhibition of tumor growth, the induction of apoptosis and the sensitization of tumors to the chemotherapy agent doxorubicin. eEF-2K down-modulation *in vitro* resulted in a decrease in the expression of c-Myc and cyclin D1 with a concomitant increase in the expression of p27^Kip1^. A decrease in the basal activity of c-Src (phospho-Tyr-416), focal adhesion kinase (phospho-Tyr-397), and Akt (phospho-Ser-473) was also detected following eEF-2K down-regulation in MDA-MB-231 cells, as determined by Western blotting. Where tested, similar results were seen in ER-positive MCF-7 cells. These effects were also accompanied by a decrease in the observed invasive phenotype of the MDA-MB-231 cells. These data support the notion that the disruption of eEF-2K expression in breast cancer cells results in the down-regulation of signaling pathways affecting growth, survival and resistance and has potential as a therapeutic approach for the treatment of breast cancer.

## Introduction

Breast Cancer is the most common malignancy in women in the Western world. Every year about 200, 000 women are diagnosed with breast cancer in the US, and more than 40,000 die from this disease. Despite the fact that combination chemotherapy regimens elicit a 50–70% objective response rate in patients with metastatic breast carcinoma, less than 20% of patients achieve durable complete remission [1]. The major reason for patient death is due to metastasis and resistance to current therapies that include chemotherapy, hormonal therapy and radiation [2]. Thus, the development of novel targeted therapeutic strategies is urgently needed to enhance the efficacy of current therapies and prolong patient survival.

In recent years eukaryotic elongation factor 2 kinase (eEF-2K) has garnered interest as a potential cancer-therapeutic target [3]. eEF-2K is an unusual calcium/calmodulin (Ca^2+^/CaM)-dependent Ser/Thr-kinase that is subject to extensive regulation by post-translational phosphorylation [4], [5]. It phosphorylates elongation factor 2 (eEF2), an event associated with decreased global protein translation [6]. Increased eEF-2K activity has been reported in breast cancer specimens, but is absent in normal adjacent tissue [7]. It has also been shown to be active in proliferating cells (e.g. breast cancer cells [8], malignant glioma cells [9], and HL60 leukemia cells [10]), whereas non-proliferating cells are reported to exhibit low levels of activity [11]. It has been suggested that progression through the G1-phase of the cell cycle and entry into the S phase (the G1/S transition) is promoted by eEF-2K activity [11], which is mediated by a rise in intracellular calcium (Ca^2+^) [12]. However, not all the reported evidence supports a role for eEF-2K in promoting proliferation. For example, a recent assessment of eEF-2K by siRNA-mediated knockdown as well as by a moderately potent ATP-competitive inhibitor of eEF-2K suggested that the contribution of eEF-2K to the proliferation of several common cancer cell lines is minimal [13]. Interestingly, mice lacking eEF-2K are reported to be normal [14]. Unfortunately, a comparison of the rate of proliferation of fibroblasts between wild type fibroblasts and those derived from mice lacking eEF-2K has not been reported.

Hypoxia, nutrient deprivation and metabolic stress are all known to stimulate eEF-2K through activation of AMPK [15]. Thus, activating eEF-2K may be a survival strategy associated with conserving ATP consumption associated with protein synthesis [16]. However, this view is complicated by the observation that hypoxia in transformed breast cancer cells may not lead to an inhibition of protein translation [17]. It has been suggested that eEF-2K mediates cytoprotection to some anti-cancer therapies through the up-regulation of autophagy [3]. Yang and colleagues have demonstrated a cytoprotective role for eEF-2K in glioma [18]–[23] and breast cancer cells [24]. They have shown that its down-regulation potentiates the effects of glucose deprivation [20] and tumor necrosis factor-related apoptosis-inducing ligand (TRAIL) [23] in glioma cells, and the inhibition of growth signaling in breast cancer cells [24]. It has also been implicated as a component of the ER stress response [25], however this needs further delineation.

Progress in understanding the physiological functions of eEF-2K and assessing it as a therapeutic target in appropriate *in vivo* cancer models have been hampered by the lack of potent pharmaceutical agents selective for eEF-2K [13], [26]. In the present study we employ small interfering RNA (siRNA). siRNA has become an important tool to knock-down gene expression and has potential for use as a targeted therapeutic modality for cancer [27]. siRNA specifically binds target mRNA and causes its degradation, inhibiting protein expression. However, *in vivo* delivery of siRNA-based therapeutics efficiently and specifically to a primary tumor and its metastases remains a great challenge. While negatively charged cell membranes prevent efficient intracellular delivery of nucleotides, cationic liposomes exhibit toxicity to mammalian cells [28]. In this study, we employ a recently developed approach using neutral nanoliposomes (∼mean diameter 65 nm) composed of neutral-charge dioleyl phosphatidylcholine (DOPC) [29] to investigate the effect of *in vivo* therapeutic targeting of the eEF-2K gene by systemic delivery of liposomal siRNA. Using this approach we can target siRNA into tumor cells *in vivo* 10- and 30-fold more effectively than cationic liposomes and naked siRNA, respectively [29].

We show that targeting eEF-2K expression by neutral liposomal siRNA results in depletion of both eEF-2K and phosphorylated eEF2 and leads to inhibition of the growth of triple negative MDA-MB-231 tumors, a highly aggressive and metastatic tumor type, in an orthotopic nude mouse model of breast cancer. We also show that this targeting provides a notable enhancement to the anti-tumor efficacy of doxorubicin, a common agent used in chemotherapy regimens, as determined by increased growth inhibition and apoptosis compared to a treatment with doxorubicin alone. *In vitro* studies aimed at revealing molecular mechanisms underlying the effect of eEF-2K down-regulation suggest that eEF-2K maintains the expression of several pro-tumorigenic proteins in breast cancer cells (e.g. c-Myc and cyclin D1), and is important for the constitutive activity of c-Src, focal adhesion kinase and Akt. Taken together, our data indicate that targeting eEF-2K in combination with chemotherapy may be a therapeutic strategy for the treatment of breast cancer.

## Materials and Methods

### Cell lines, culture conditions and reagents

The human breast cancer cell lines employed were obtained from American Type Culture Collection (Manassas, VA). MCF-7/R (drug resistant) cells were a gift from Dr. Kapil Mehta, Ph.D. (M. D. Anderson Cancer Center, Houston, TX). All cell lines were cultured in DMEM/F12 supplemented with 10% FBS, except SK-BR3 (RPMI supplemented with 10% FBS) and MCF-10A (DMEM/F12 supplemented with 5% horse serum, EGF, hydrocortisone, insulin and Cholera toxin). Cells were cultured at 37°C in a humidified incubator with 5% CO_2_.

### Plasmids

*EGB2T-eEF-2K* – cDNA from the bacterial expression vector *p32TeEF-2K*, encoding the human Trx-His_6_-tagged eEF-2K (GenBank accession number NM_013302), was used as a template in a PCR reaction [30]. To clone the human eEF-2K cDNA, the desired sequence was amplified by PCR using a specifically designed forward primer, *5′-* TTT GGT ACC ATG GCA GAC GAA GAT CTC ATC-*3′* (*KpnI* recognition site underlined) and reverse primer, *5′* -AAA TGC GGC CGC TTA CTC CTC CAT CTG GGC CCA-*3′* (*NotI* recognition site underlined), and ligated into the EGB-2T vector [31].

### Western blot analysis

Following treatments, the cells were washed twice in ice cold PBS and lysed at 4°C. Protein concentration for each sample was determined by Bradford assay (Bio-Rad, Hercules, CA), and Western blotting was performed. The membranes were blocked with 5% dry milk or BSA and probed with the following primary antibodies: eEF-2K, p-EF2 (Thr-56), EF2, cyclin D1, p27, p-Akt (Ser-473), Akt, p-Src (Tyr-416), Src, p-paxillin (Tyr-31), p-mTOR (Ser-2448), mTOR (Cell Signaling Technology, Danvers, MA); HIF1α, p-FAK (Tyr-397), FAK (BD Transfection); c-Myc, Bcl-2 and caspase-9 (cleaved) (Santa Cruz Biotechnology, Santa Cruz, CA). Horseradish peroxidase-conjugated anti-rabbit or anti-mouse secondary antibody (Amersham Life Science, Cleveland, OH) were used for detection. Chemiluminescent detection was performed with Chemi-glow detection reagents (Alpha Innotech, San Leandro, CA). The blots were visualized with a FluorChem 8900 imager and quantified by a densitometer using the Alpha Imager application program (Alpha Innotech, San Leandro, CA). Mouse anti-β-actin (primary) and donkey anti-mouse (secondary) antibodies (Sigma Chemical, St. Louis, MO) were used to monitor β-actin expression as a loading control.

### Transfections with siRNA and plasmid

siRNA targeting eEF-2K was designed using siRNA-designing software (Qiagen): eEF-2K siRNA#1, *5′*-GCCAACCAGUACUACCAAA-*3′*. A previously published eEF-2K siRNA: eEF-2K siRNA#2, *5′*-AAGCUCGAACCAGAAUGUC-*3′* [3], control non-silencing siRNA (*5′*-AAUUCUCCGAACGUGUCACGU-*3′*) [32] and siRNA targeting c-Src (Sigma-Aldrich, St. Louis, MO) were also employed. Cells were transfected with siRNA as previously described. siRNA (1 µg/well) or GST-eEF-2K plasmid (1–3 µg/well) were transfected using HiPerFect Transfection Reagent (Qiagen, Valencia, CA) and Qiagen Plasmid Transfection Reagent respectively, according to the manufacturer's protocol.

### Cell viability and proliferation/growth assays

Viable and dead cells were detected by the trypan blue exclusion assay. Additionally, the MTS assay was used to detect the viability and/or proliferation of cells. All experiments were performed in triplicate.

### Clonogenic survival assay

Briefly, cells were transfected with control or eEF-2K siRNA every week and grown for 2 to 3 weeks. Cells were stained with crystal violet and colonies were counted. Each experiment was performed in duplicate [33].

### Analysis of cell death

Apoptosis was assessed by an Annexin V assay, as well as by monitoring caspase 9 cleavage by Western blotting. Cells were treated with eEF-2K siRNA or control siRNA for 24 to 96 hours, and then analyzed by Annexin V and propidium iodide (PI) staining according to the manufacturer's protocol (BD Pharmingen Annexin V kit, San Diego, CA). Positive cells were detected and quantified by FACS analysis.

### Matrigel invasion assay

MDA-MB-231 cells were transfected with control or eEF-2K siRNA, and 24 h later, cells were seeded onto Matrigel-coated Transwell filters (8-µm pore size) in Matrigel invasion chambers (BD Biosciences, San Jose, CA). The number of cells that invaded the lower side of the membrane was determined at 72 h by counting cells in a minimum of four randomly selected areas.

### Liposomal siRNA preparation

For *in vivo* delivery, siRNA was incorporated into dioleoyl-sn-glycero-3-phosphocholine (DOPC). DOPC and siRNA were mixed in the presence of excess tertiary butanol at a ratio of 1∶10 (*w/w*) siRNA/DOPC [34]. Before *in vivo* administration, the preparation was hydrated with normal 0.9% saline (100 µL per mouse) for *i.v.* injection.

### Orthotopic xenograft tumor model of breast cancer

Athymic female nu/nu mice (5 week old) were obtained from the Department of Experimental Radiation Oncology at M. D. Anderson Cancer Center, Houston, TX. All studies were conducted according to an experimental protocol approved by the M. D. Anderson Institutional Animal Care and Use Committee. MDA-MB-231 cells (1×10^6^) were injected into the right middle mammary fat pad of each mouse. Two weeks after injection, when tumor size reached about 3–5 mm, liposomal siRNA treatments were initiated. Each mouse received 150 µg/kg (equivalent of ∼4 µg/mouse) non-silencing control siRNA or eEF-2K siRNA twice a week (*i.v.* injection into the tail vein in 100 µL saline) for four weeks. After completion of treatments, mice were euthanized with CO_2_. Tumor tissues were removed for Western blot, Immunohistochemistry and TUNEL analysis, and weighed to measure tumor growth.

### Ethics statement

This study was performed in strict accordance with the recommendations in the Guide for the Care and Use of Laboratory Animals of the National Institutes of Health. The protocol was approved by The University of Texas at Austin Institutional Animal Care and Use Committee (Permit Number: 11062701).

### TdT-mediated dUTP nick end labeling (TUNEL) assay

Apoptotic events after treatment with liposomal siRNA and/or chemotherapy regimens were determined by the TdT-mediated dUTP nick end labeling (TUNEL) colorimetric assay (Promega, Madison, WI) in tumor sections, according to the manufacturer's protocol as described previously [35]. For each tumor section, five randomly selected fields were used to visualize cells at 400× magnification. The number of cells registering as positive in the TUNEL assay (dark brown nuclei) was recorded. Four tumor sections were analyzed for each experiment.

### Statistical analysis

The data were expressed as the means ± SD of three or more independent experiments, and statistical analysis was performed using the two-tailed and paired Student's *t*-test. *P* values less than 0.05 were considered statistically significant and are indicated by an asterisk.

## Results

### eEF-2K regulates cell growth and clonogenicity

**Figure 1:**
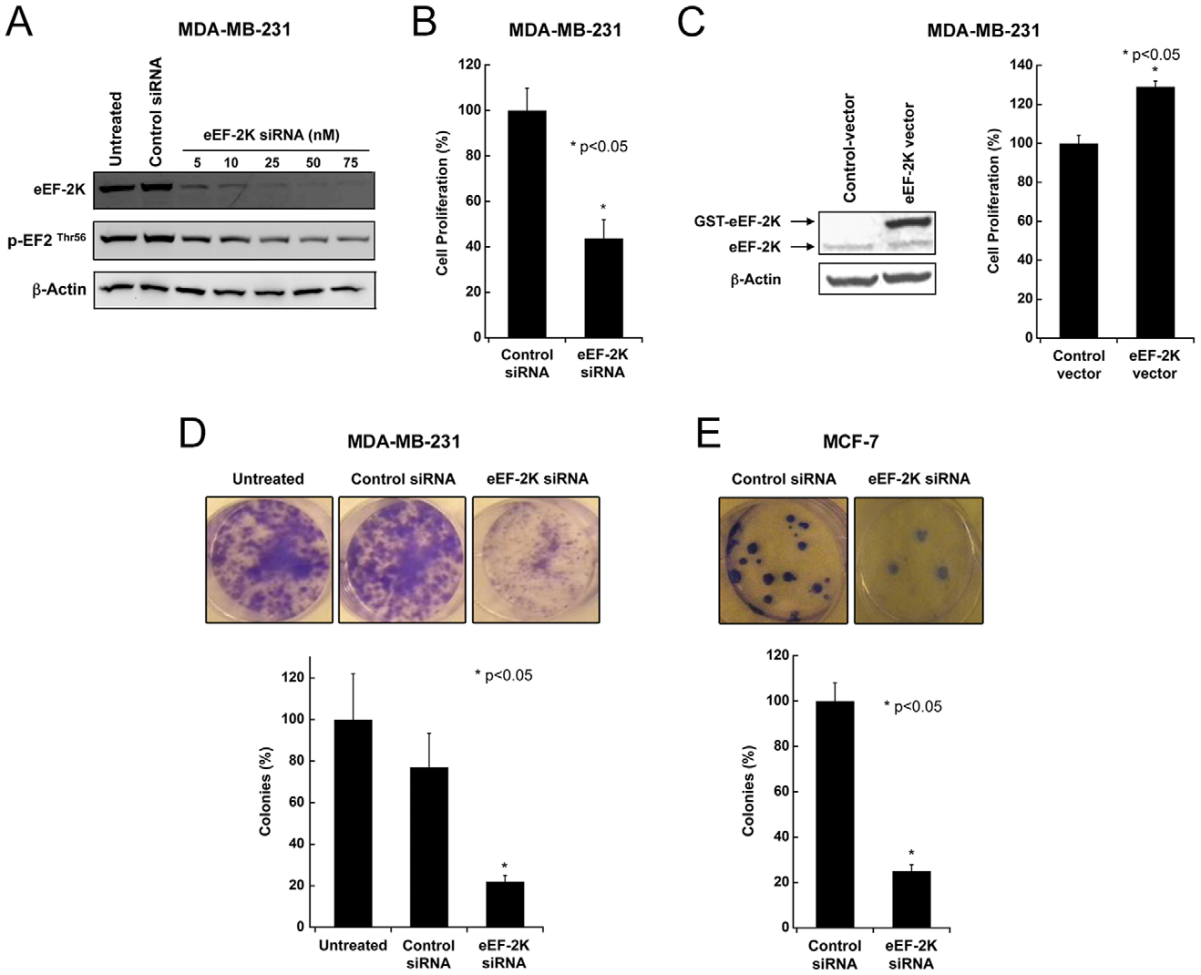
Effect of eEF-2K down-regulation on breast cancer cell proliferation and clonogenicity. (**A**) Knockdown of eEF-2K by siRNA. siRNA specifically targeting eEF-2K inhibits its expression in MDA-MB-231 cells. Cells were transfected with eEF-2K siRNA (5–75 nM) for 48 h, and cell lysates were subjected to Western blot analysis. (**B**) Effect of knockdown of eEF-2K on cell proliferation. MDA-MB-231 cells were transfected with eEF-2K siRNA, and after 48 h proliferation was detected by an MTS assay. (**C**) Overexpression of eEF-2K in MDA-MB-231 cells. Cells were transiently transfected with an expression vector encoding GST-tagged wild type eEF-2K, and cell lysates (48 h) were subjected to Western blot analysis. For the effect of overexpression of eEF-2K on cell proliferation, MDA-MB-231 cells were transiently transfected with eEF-2K or a vector control, and cell proliferation was measured using an MTS assay after 96 h. (**D–E**) Effect of eEF-2K knockdown on colony formation. Knockdown of eEF-2K by siRNA (50 nM) significantly inhibited the number of colonies formed by MDA-MB-231 (D) and MCF-7 (E) cells (*p<0.05). Cells were transfected every 4 days with control or eEF-2K siRNA.

The purpose of this study was to evaluate the potential of targeting the protein kinase eEF-2K in breast tumors, with the objectives of determining whether targeting eEF-2K 1) impedes tumor growth, and 2) sensitizes tumors to chemotherapy. Early studies using the non-specific compound rottlerin suggested that eEF-2K is important for the proliferation of breast cancer cells [11]. However, recent *in vitro* studies have been contradictory, both refuting [13], and supporting these original observations [24]. Therefore, before evaluating the targeting of eEF-2K *in vivo*, we decided to re-evaluate it *in vitro* using both an MTS cell proliferation assay as well as a clonogenic assay. As Rottlerin is known to inhibit many other signaling pathways, and potent and selective inhibitors of eEF-2K are not available, we decided to down-regulate the expression of eEF-2K using an siRNA approach. We designed appropriate siRNAs and assessed their ability to down-regulate eEF-2K expression in MDA-MB-231 and MCF-7 cells. The characterization of these cells has been described [36]. MDA-MB-231 are ER-negative and highly invasive, whereas MCF-7 are ER-positive and minimally invasive. As shown in Figure 1A, 50 and 75 nM of an siRNA targeting eEF-2K (eEF-2K siRNA#1) efficiently decreased eEF-2K expression by about 90% or more 48 h after transfecting MDA-MB-231 cells. As expected [37], inhibition of eEF-2K by siRNA resulted in a reduction in the phosphorylation of eEF2 on Thr-56 (Figure 1A). Similar effects were observed in MCF-7 cells (see Figure S1A where a second siRNA, eEF-2K siRNA#2 was utilized).

The effect of down-regulating eEF-2K by siRNA was then examined in MDA-MB-231 cells using an MTS assay to determine cell proliferation during log phase of growth. Figure 1B reveals that eEF-2K down-regulation results in a 2-fold reduction in cell count, compared to the control, following 72 hours of proliferation. A similar result was obtained using MCF-7 cells (data not shown). eEF-2K siRNA#2 exhibited similar results in three breast cancer cell lines compared to the appropriate control cells (Figure S2). To further evaluate the sensitivity of breast cancer cells to eEF-2K, we transiently transfected MDA-MB-231 cells with GST-eEF-2K using an expression vector encoding for wild type eEF-2K (Figure 1C). The overexpression of eEF-2K resulted in a modest 1.2-fold increase in the number of cells in the assay compared to control cells, which were transfected with an empty vector (Figure 1C).

We then determined the effects of targeting eEF-2K on colony formation (clonogenicity) in MDA-MB-231 and MCF-7 cells. This assay measures the ability of tumor cells to grow and form foci [33]. In this assay, normal cells become growth contact inhibited, thus the assay is a measure of the propensity of cancer cells to undergo neoplastic transformation. Clonogenicity was determined by plating cells into tissue culture dishes at a fixed number, and culturing for 14 days. When MDA-MB-231 and MCF-7 cells were transfected with either eEF-2K or control siRNA (50 nM) every 4 days over a period of 14 days, pronounced reduction in colony formation of both MDA-MB-231 (Figure 1D) and MCF-7 (Figure 1E and S3) cells was observed in eEF-2K-targeted cells compared to control cells..

### eEF-2K regulates the expression of cyclin D1 and p27^Kip1^

**Figure 2:**
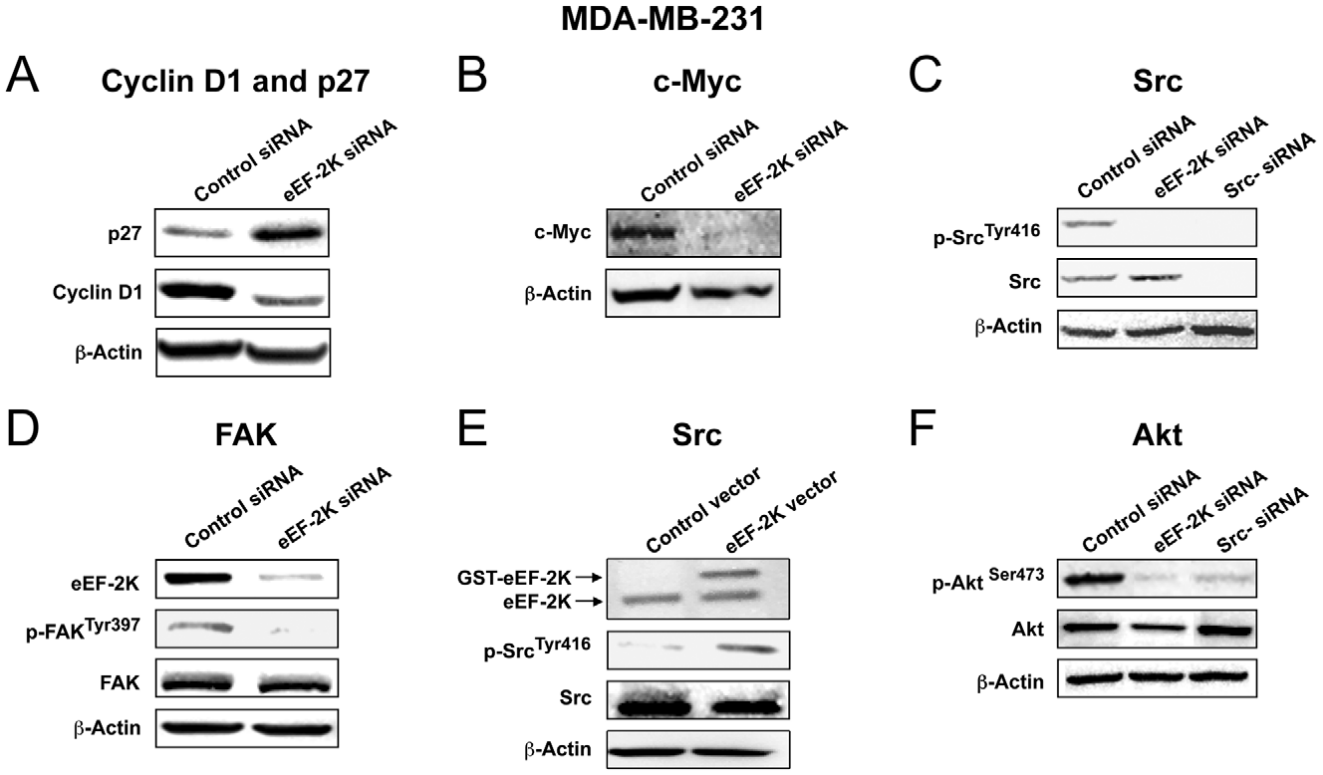
Downstream molecular effects of knockdown or overexpression of eEF-2K in MDA-MB-231 cells. Cells were transiently transfected with eEF-2K siRNA or GST-eEF-2K plasmid, and cell lysates (72 h) were subjected to Western blot analysis. (**A–B**) Knockdown of eEF-2K by siRNA increases expression levels of p27^Kip1^ (A), while decreasing cyclin D1 (A) and c-Myc (B) levels. (**C–D**) eEF-2K knockdown additionally inhibits the activity of Src and FAK as indicated by reduced p-Src (Tyr-416) (C) and p-FAK (Tyr-397) (D). (**E**) Conversely, overexpression of eEF-2K resulted in increased p-Src (Tyr-416) levels. (**F**) Knockdown of eEF-2K also inhibits the activity of Akt as indicated by reduced p-Akt (Ser-473).

As eEF-2K has been shown to exhibit high activity during the S-phase of the cell cycle [38], [39], and eEF-2K knockdown reduced cell proliferation (Figure 1), we examined whether its down-regulation affected the regulation of cyclin D1 and p27^Kip1^ cyclin-dependent kinase inhibitor (CKI) which are known to regulate G1 to S phase progression. Western blot analysis revealed that knockdown of eEF-2K markedly reduced cyclin D1 expression and increased p27^Kip1^ expression in MDA-MB-231 cells (Figure 2A and S1B). The tumor suppressor PDCD4, which we have previously shown to up-regulate p27^Kip1^ expression [40], was also induced upon eEF-2K down-regulation (data not shown). These data support the notion that eEF-2K promotes progression of MDA-MB-231 cells through the G1/S transition of the cell cycle.

### eEF-2K regulates the expression of c-Myc

As c-Myc is known to be expressed in MDA-MB-231 cells and regulates cyclin D1 [41] and p27^Kip1^ [42], we wondered whether eEF-2K may regulate c-Myc. As shown in Figure 2B, eEF-2K knockdown significantly reduced c-Myc expression in MDA-MB-231 cells compared to cells treated with control siRNA. A similar effect was also observed in MCF-7 cells using the two previously described siRNAs (Figure S1C).

### Down-regulating eEF-2K expression using systemically administered liposomal siRNA inhibits orthotopic tumor growth in a breast cancer model

**Figure 3:**
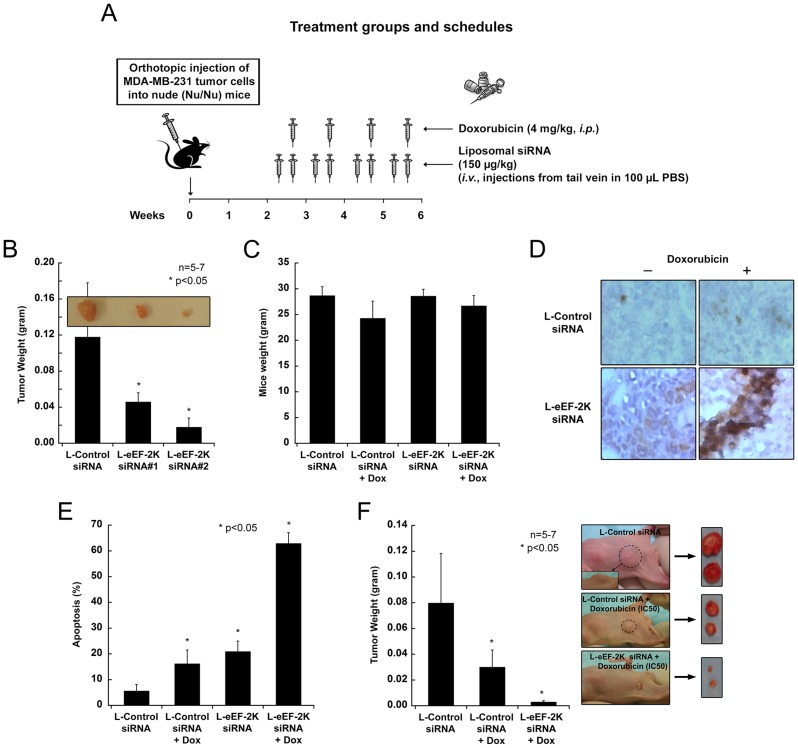
Systemic *in vivo* targeting of eEF-2K by liposomal siRNA. (**A**) Treatment schedule of nude mice bearing MDA-MB-231 tumors with liposomal siRNA (control and eEF-2K) and/or doxorubicin. About 2 weeks after tumor cell injection (MDA-MB-231 cells), targeting of eEF-2K was achieved by systemic (*i.v.* from tail vein) administration of DOPC-liposomal eEF-2K siRNA (150 µg/kg or about 4 µg/mouse) twice a week and/or doxorubicin once a week (*i.p.*, 4 mg/kg), for 4 weeks. (**B**) Tumor weight of L-Control and L-eEF-2K siRNA treated mice. Therapeutic targeting of the eEF-2K gene was achieved by systemically administering (*i.v.* twice a week, 150 µg/kg) DOPC-liposomal siRNA (L-eEF-2K siRNA#1 and siRNA#2) in nude mice bearing MDA-MB-231 tumors. Non-silencing DOPC-liposomal siRNA (L-Control siRNA) was used as a control. After 4 weeks of treatment, tumor weight was measured. (**C**) The mean weight of mice after four weeks of treatment. No toxic effects were observed in mice exposed to liposomal eEF-2K siRNA ± doxorubicin for four weeks, compared with the control group. Mice appeared healthy and did not lose weight during treatment. (**D–E**) TUNEL assay indicating apoptosis (D) and quantification (% apoptosis) of TUNEL assay (E) in tumors in mice treated with L-eEF-2K siRNA and/or doxorubicin. Brown staining in the TUNEL assay is a positive indicator of apoptosis. (**F**) *In vivo* targeting of eEF-2K enhances the efficacy of chemotherapy. Mice bearing MDA-MB-231 tumors were given L-eEF-2K siRNA or L-Control siRNA (*i.v.* twice a week, 150 µg/kg) and doxorubicin once a week (*i.p.*, 4 mg/kg) for 4 weeks, after which tumor weights were measured.

**Figure 4:**
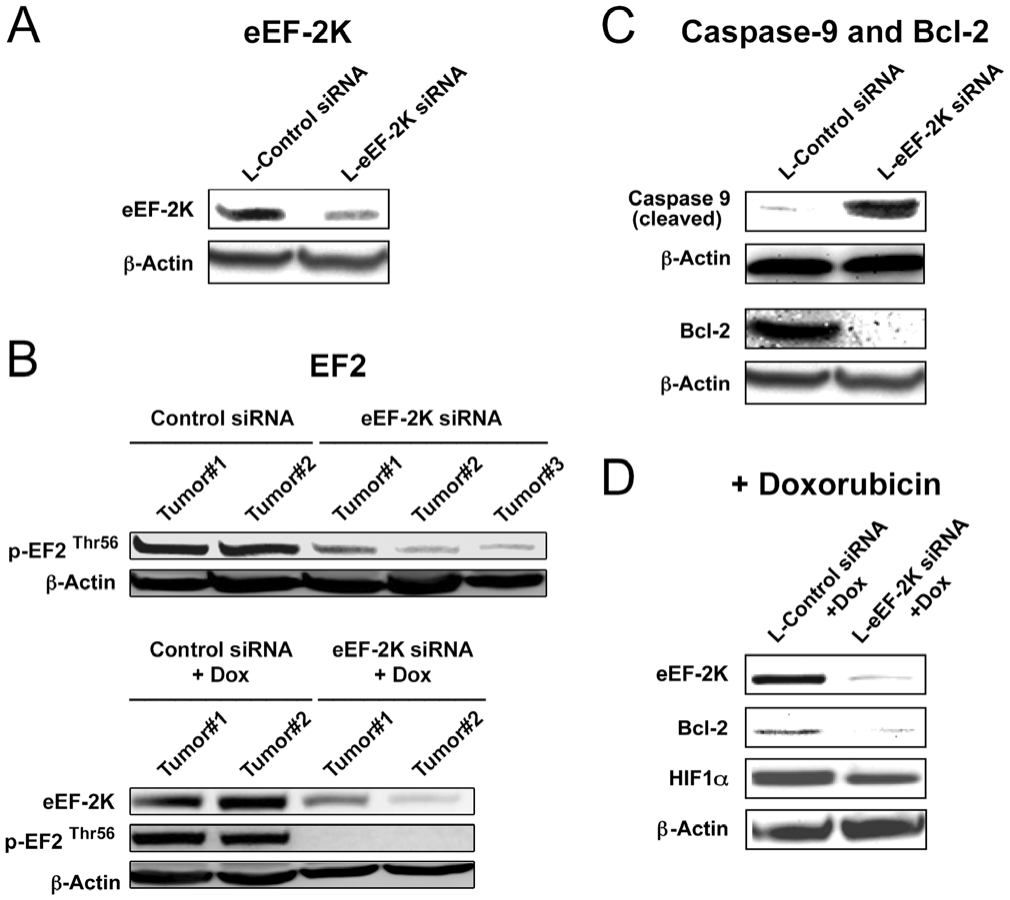
Molecular effects of *in vivo* targeting of eEF-2K by liposomal siRNA. Following treatment with L-eEF-2K siRNA, tumor tissues were removed from mice and subjected to Western blot analysis. (**A–B**) Treatment of mice with L-eEF-2K siRNA results in the knockdown of eEF-2K in different tumors (A–B), with the consequent reduction in p-eEF2 (Thr-56) levels (B). (**C**) L-eEF-2K siRNA treatment induces apoptosis in tumors as indicated by caspase-9 cleavage and a decrease in anti-apoptotic Bcl-2 levels. (**D**) Depletion of eEF-2K enhances doxorubicin-induced Bcl-2 and HIF1α down-regulation.

We investigated the therapeutic potential of targeting eEF-2K systemically in an orthotopic model of breast cancer (MDA-MB-231) in nude mice [43], [44]. Given the previous success of our therapeutic siRNA delivery strategy, which utilizes a delivery of low doses of siRNA to tumors [44], the two siRNAs described above (siRNA#1 and siRNA#2) were used to target eEF-2K [3]. This was achieved by systemic (*i.v.*) administration (tail vein) of DOPC-liposomal eEF-2K siRNA (150 µg/kg or about 4 µg/mouse) twice a week for 4 weeks (Figure 3A). Treatment with both liposomal eEF-2K siRNA#1 and siRNA#2 resulted in significant down-regulation of its expression in the tumors (Figure 4A & 4B), a decrease in phosphorylated eEF2 (Figure 4B) and significant inhibition of tumor growth (Figure 3B). No toxic effects were observed in the mice exposed to liposomal eEF-2K siRNA for four weeks compared with the control group: the mice appeared healthy and did not lose weight during the treatment. The mean weight of those treated with L-eEF-2K siRNA and those treated with the L-control siRNA was 28.3±0.8 g and 28.4±0.4 g, respectively (Figure 3C). Given the pronounced growth inhibitory effects of down-regulating eEF-2K, the tumor samples were examined for evidence of the induction of apoptosis. Examination of tumor tissue from L-eEF-2K siRNA-treated mice revealed significant cleavage of caspase-9 (Figure 4C), a positive TUNEL assay (brown staining) (Figure 3D and 3E) and the down-regulation of the anti-apoptotic protein Bcl-2 (Figure. 4C). These data show, for the first time, that eEF-2K knockdown has inhibitory effects on tumor growth in an *in vivo* orthotopic model (Figure 3B), which is associated with an increase in the apoptosis of tumor cells.

### Targeting of eEF-2K enhances the efficacy of chemotherapy both *in vivo* and *in vitro*

Recently, knocking down eEF-2K in glial cells was shown to increase cellular apoptosis following treatment with the Akt inhibitor MK-2206 in a non-orthotopic xenograft model [21]. In this experiment, glial cells were first treated with siRNA before injection of the cells into mice. Several *in vitro* studies have also pointed to a benefit from down-regulating eEF-2K. For example, its down-regulation is reported to sensitize glioma cells to TRAIL [23] and 2-deoxy-D-glucose [20], and to augment the effect of inhibiting growth factor receptor signaling in breast cancer cells [24]. Doxorubicin is an anthracycline antibiotic, and an important agent in a number of chemotherapy regimens. While considered to be one of the most effective agents for breast cancer treatment, resistance is almost universally encountered [45]. Given the findings of Yang and collaborators [18]–[24], we decided to examine the effect of *systemically* down-regulating eEF-2K on the sensitivity of established tumors to doxorubicin in the orthotopic model. Thus, a group of mice were orthotopically injected with MDA-MB-231 cells as described above. Two weeks after tumor inoculation, mice were given DOPC-L-eEF-2K siRNA twice a week as described above and doxorubicin once a week (*i.p.*, 4 mg/kg) for 4 weeks (Figure 3A). Figure 3F shows that the group receiving combination therapy (L-eEF-2K siRNA + doxorubicin) had the smallest tumors (p<0.05) compared to L-control siRNA or L-control siRNA + doxorubicin groups. In addition, eEF-2K knockdown in tumors enhanced the doxorubicin-mediated inhibition of expression of the pro-survival protein Bcl-2 as well as the hypoxia-related transcription factor HIF1α (Figure 4D). These findings show, for the first time in an orthotopic model, that the silencing of eEF-2K may significantly enhance the effect of doxorubicin against breast cancer.

**Figure 5:**
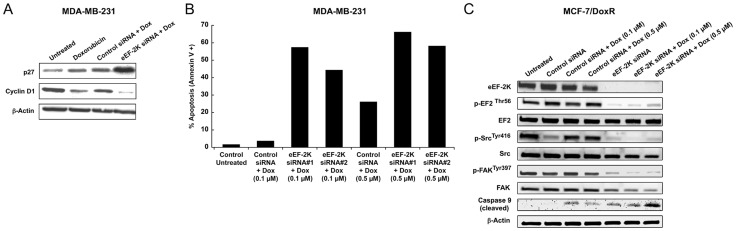
Effect of down-regulation of eEF-2K by siRNA on doxorubicin efficacy against breast cancer cells. (**A**) Western blot analysis demonstrated that knockdown of eEF-2K in doxorubicin-treated MDA-MB-231 cells further reduced cyclin D1 levels while increasing p27^Kip1^ levels. (**B**) Knockdown of eEF-2K using two different siRNA enhances efficacy of doxorubicin-induced (0.1 and 0.5 ìM) apoptosis of MDA-MB-231 breast cancer cells. Apoptosis was detected by Annexin V staining and the percentage of positive cells was quantified by FACS analysis after 48 h of doxorubicin treatment. (**C**) Down-regulation of eEF-2K enhances inhibition of Src and FAK activity and increases caspase-9 cleavage in doxorubicin-treated MCF-7/DoxR cells.

In *in vitro* studies, we showed that eEF-2K knockdown further increases doxorubicin-mediated induction of p27^Kip1^ expression (Figure 5A) and significantly enhances doxorubicin-induced apoptosis in MDA-MB-231 cells, where inhibition of eEF-2K dramatically increases doxorubicin-induced apoptosis from 5% to 55% (Figure 5B). To further evaluate the ability of eEF-2K knockdown to sensitize breast cancer cells to doxorubicin, we decided to assess whether eEF-2K knockdown sensitized MCF-7 cells that are resistant to doxorubicin (MCF-7/DoxR cells). The cells were treated with either eEF-2K siRNA (50 nM) or control siRNA (50 nM) for 24 hours, followed by doxorubicin for 48 hours. Figure 5C shows that the down-regulation of eEF-2K in these cells significantly enhances caspase-9 cleavage, indicative of increased apoptosis.

**Figure 6:**
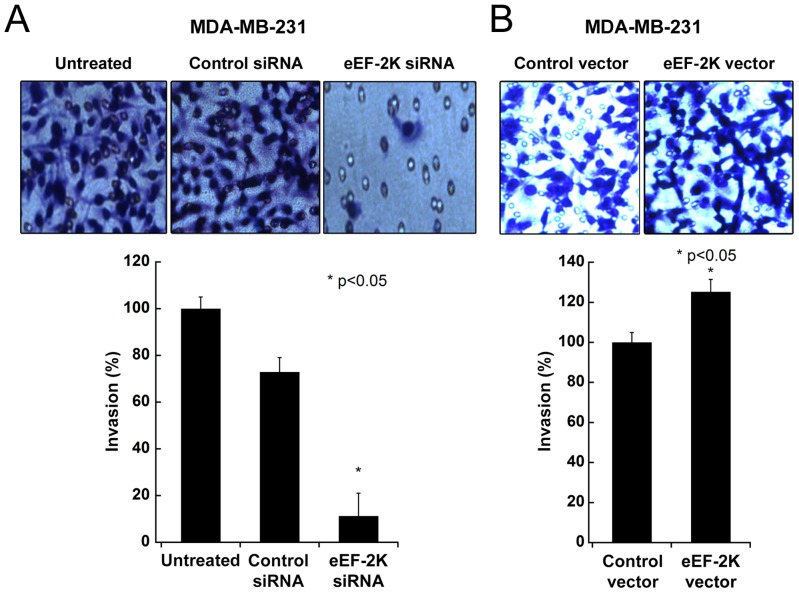
Effect of knockdown or overexpression of eEF-2K on invasion of MDA-MB-231 cells. Depletion of eEF-2K by siRNA inhibits invasion (A), while overexpression of eEF-2K increases invasion (B) of MDA-MB-231 cells in Matrigel (72 h).

### Targeting eEF-2K impairs invasion of breast cancer cells

As eEF-2K is reported to play a role in the invasiveness of human glioma cells [22], we wondered whether it might also regulate the invasiveness of MDA-MB-231 breast cancer cells, which have a highly invasive phenotype. Therefore, *in vitro* invasion assays were performed using Matrigel-coated Boyden chambers. This assay mimics the *in vivo* invasion process and measures the number of cancer cells passing through a basement membrane matrix (Matrigel) towards media containing chemo-attractants [46]. The depletion of eEF-2K by siRNA reduced invasion of MDA-MB-231 cells by 6-fold (Figure 6A). Notably, its overexpression also slightly increased invasion by 1.2-fold (Figure 6B). These data suggest that eEF-2K supports breast cancer cell invasion and that its inhibition may reduce the invasion and metastatic potential of breast cancer cells. It should be noted that 20% or less of the cells were positive in an Annexin V assay (data not shown), suggesting that some, but not all, of the observed effects on invasion are due to an increased population of cells undergoing apoptosis.

**Figure 7:**
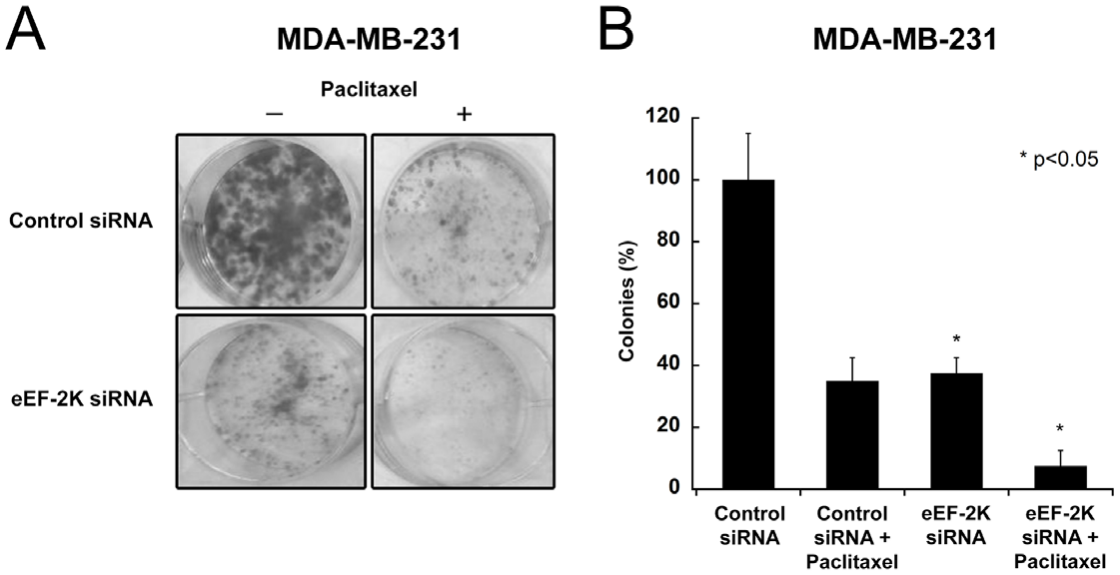
Knockdown of eEF-2K enhances the inhibitory effect of paclitaxel on MDA-MB-231 cells. Down-regulation of eEF-2K by siRNA enhances the efficacy of paclitaxel-induced (2 nM) inhibition of colony formation in highly aggressive and metastatic MDA-MB-231 breast cancer cells. (**A**) Cell colonies stained with crystal violet and (**B**) Quantification of colony formation represented graphically.

**Figure 8:**
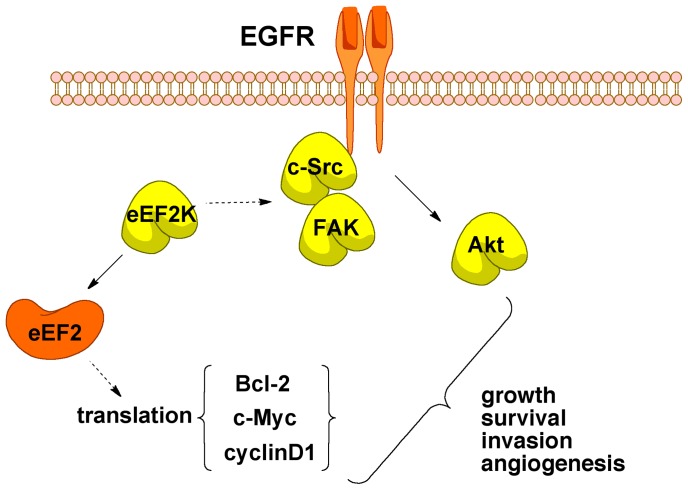
Targeting eEF-2K disrupts signal transduction pathways that promote tumorigenesis in breast cancer cells. The phosphorylation of eEF2 by eEF-2K leads to an increase in the phospho-eEF2/eEF2 ratio in transformed breast cancer cells, which is predicted to influence protein translation, possibly facilitating the selective translation of mRNAs possessing structured *5′*-UTRs encoding for proteins such as c-Myc, Bcl-2 and cyclin D1. eEF-2K facilitates basal c-Src activation through a mechanism that is currently unknown. c-Src is an important mediator of many downstream effects of receptor tyrosine kinases, including the EGFR family, and mediates basal activation of Akt by eEF-2K. Pathways facilitated by eEF-2K are implicated in cell growth, survival, motility and angiogenesis, supporting the notion that it may be a potential cancer therapeutic target. Known mechanisms are represented as solid arrows. Potential mechanisms are represented as dashed arrows.

### Targeting eEF-2K impairs basal c-Src and focal adhesion kinase activity

To elucidate potential molecular mechanisms by which eEF-2K influences invasion, we examined the possible involvement of the non-receptor protein tyrosine kinase c-Src. The Src family of non-receptor protein tyrosine kinases are known to play critical roles in adhesion, migration and invasion as well as proliferation in breast cancer cells [47], and c-Src is of considerable interest as a molecular target for treatment [48]. c-Src is an important mediator of many downstream effects of receptor tyrosine kinases, including the EGFR family, and is overexpressed and constitutively active in MDA-MB-231 cells [49]. EGFR1 (HER1) is also overexpressed in these cells and its activity is potentially enhanced by c-Src [49], [50]. c-Src activation is associated with the phosphorylation of Tyr-416 and dephosphorylation at the negative regulatory site Tyr-527 [51]. Figure 2C shows that knockdown of eEF-2K by siRNA led to a significant reduction in c-Src phosphorylated at Tyr-416 in MDA-MB-231 cells. Focal adhesion kinase (FAK) regulates cell adhesion and migration, and is autophosphorylated on Tyr-397, which results in increased kinase activity upon engagement with integrins [52]. Figure 2D shows that a significant reduction in FAK phosphorylated on Tyr-397 accompanies the observed decrease in c-Src activation, consistent with the known cooperativity between FAK and c-Src in cancer cells [53]. Notably, the overexpression of eEF-2K resulted in a significant increase in c-Src phosphorylated at Tyr-416 (Figure 2E) (as well as phospho-eEF2 (Thr-56), data not shown). These data suggest that eEF-2K may contribute to the activation of both c-Src and FAK in MDA-MB-231 cells, thereby contributing to the invasive phenotype. Similar effects of eEF-2K down-regulation were observed on c-Src/FAK activation in other cell lines, including BT20 (Figure S1D), MDA-MB-435 (Figure S1E) and doxorubicin resistant MCF-7 (MCF-7/DoxR) (Figure 5C) breast cancer cells, suggesting that this is a common mechanism of regulation in transformed breast cancer cells. eEF-2K knockdown also led to a reduction in the phosphorylation of paxillin on Tyr-31 (a target for FAK) in MCF-7/DoxR cells (data not shown), further supporting a link between eEF-2K and cytoskeletal rearrangements.

### Targeting eEF-2K impairs basal Akt activity mediated by c-Src

c-Src may regulate Akt activity through several mechanisms, including an EGFR-mediated pathway. For example, Gefitinib, an EGFR inhibitor, is reported to suppress Akt activation (as well as c-Myc expression) in MDA-MB-231 cells [54]. Activation of Akt requires the phosphorylation of Ser-473 by mTORC2 [55]. We observed a decrease in the level of Akt phosphorylated on Ser-473 upon targeting either c-Src or eEF-2K by siRNA (Figure 2F), consistent with the notion that an active eEF-2K/c-Src/Akt pathway is present in these cells.

## Discussion

### eEF-2K exhibits variable expression in transformed breast cancer cells and promotes growth and clonogenicity

Mechanisms regulating the activity of eEF-2K in breast cancer cells are not well understood. Its expression is regulated through ubiquitin-mediated proteolysis [56] and association with Hsp90 [18]. Compared to its expression in the spontaneously immortalized human breast epithelial cell line MCF-10A, its expression in several human breast carcinoma cell lines is high (reference [17] and our unpublished data). While fibroblasts derived from mice lacking eEF-2K are reported to lack phosphorylation on eEF2 [25], mice expressing a severely catalytically-impaired form of eEF-2K exhibit significant phosphorylation of eEF2 [37], suggesting no clear relationship between eEF-2K expression and eEF2 phosphorylation *in vivo*. While eEF2 phosphorylation is sensitive to the depletion of eEF-2K in MDA-MB-231 cells, it appears that a low level of eEF-2K can mediate significant phosphorylation of eEF2 (Figure 1A). We examined the growth-sensitivity of the two breast cancer cell lines MDA-MB-231 and MCF-7 to the depletion of eEF-2K. Expression of eEF-2K in MDA-MB-231 cells is considerably lower than in MCF-7 cells. However, both exhibit a similar reduction in growth and clonogenicity upon targeting eEF-2K.

### Therapeutic targeting of eEF-2K inhibits breast cancer tumor growth

In this study we provide the first evidence that systemic *in vivo* targeting of eEF-2K expression by liposomal siRNA inhibits growth (Figure 3B) and induces apoptosis (Figure 3D and 3E) of established tumors in an orthotopic xenograft model of a highly aggressive and metastatic breast cancer. As potent small molecule inhibitors of eEF-2K are not currently available [57], we investigated the potential of targeting eEF-2K by siRNA. The delivery of therapeutics to tumor cells and vasculature is recognized as a potential approach for the treatment of cancer [29]. Small interfering RNA (siRNA) has become an important tool to knock-down gene expression and has potential for use as a targeted therapeutic modality for cancer [27]. In this study we used a recently developed nanoliposomal approach [29] (∼mean liposome diameter 65 nm) where the liposomes are composed of neutral-charge dioleyl phosphatidylcholine (DOPC). We found that a twice-weekly dose (Figure 3A) afforded robust down-regulation of eEF-2K expression in the tumors (Figure 4A and 4B), a significant decrease in eEF2 phosphorylation (Figure 4B), and induced apoptosis (as judged by a positive TUNEL assay (brown staining) (Figure 3D and 3E), significant cleavage of caspase-9 (Figure 4C) and the down-regulation of the anti-apoptotic protein Bcl-2 (Figure. 4C)). Thus, these data show for the first time, that eEF-2K knockdown has inhibitory effects on tumor growth in an *in vivo* orthotopic model (Figure 3B), which is associated with an increase in the apoptosis of tumor cells.

We also provide the first *in vivo* data using an orthotopic model to support the notion that targeting eEF-2K may be a useful strategy as a co-therapy for breast cancer to enhance efficacy of commonly used chemotherapeutic agents, such as doxorubicin. Previous studies have reported that eEF-2K knockdown sensitizes glioma cells to TRAIL [23], 2-deoxy-D-glucose [20] and the Akt inhibitor MK-2206 [21], as well as breast cancer cells to the inhibition of growth factor receptor signaling [24]. Here, we show that knockdown of eEF-2K augments doxorubicin-induced growth inhibition of established tumors in mice (Figure 3F) and facilitates doxorubicin-induced apoptosis, as indicated by a TUNEL assay (Figure 3D and 3E) as well as by an enhanced decrease in Bcl-2 levels (Figure 4D). Similar effects were observed *in vitro*, where for example, the down-regulation of eEF-2K in doxorubicin-treated MDA-MB-231 cells heightened apoptosis, as indicated by the Annexin V assay (Figure 5B). Additionally, the inhibition of colony formation by the chemotherapeutic agent paclitaxel is enhanced by the down-regulation of eEF-2K (Figure 7). We also found that the targeting of eEF-2K sensitized doxorubicin-resistant MCF-7 (MCF-7/DoxR) cells to doxorubicin as evidenced by an increase in caspase-9 cleavage (Figure 5C).

### eEF-2K contributes to the maintenance or up-regulation of several pro-tumorigenic proteins

We showed that the *in vivo* inhibition of eEF-2K induced the down-regulation of the anti-apoptotic protein Bcl-2 [32], [58] (Figure 4C), which is notable because we have shown previously that *in vivo* targeted silencing of Bcl-2 by liposomal Bcl-2 siRNA inhibits growth of Bcl-2-expressing breast cancer tumors, including MCF-7 and MDA-MB-231 tumors, in nude mice [59]. We also found that the down-regulation of eEF-2K inhibited the expression of cyclin D1 and c-Myc, and induced the expression of p27^Kip1^ in MDA-MB-231 cells (Figure 2A and 2B). Cyclin D1 promotes entry of cells into the S-phase of the cell cycle, induces proliferation and can cause malignant transformation. The CDK-inhibitor p27^Kip1^ is a key regulator of the G1-to-S-phase progression [60] and is known to be inhibited by c-Myc [61], which is also known to regulate the expression of metabolic genes associated with metabolic reprogramming in cancer cells [62]. We found that knockdown of eEF-2K abrogates basal c-Src/FAK signaling in several cell lines (Figure 2C, 2D, S1D and S1E). c-Src is overexpressed and exhibits high basal activity in MDA-MB-231 cells, and is known to play a role in invasion and metastasis as well as proliferation [47]. In this study we showed that active c-Src is required to maintain constitutively active Akt in MDA-MB-231 cells, and that targeting eEF-2K inhibits this activity (Figure 2C and 2F).

### eEF-2K activity as a positive regulator of selective protein expression

It has been suggested that inhibiting eEF-2K blunts the ability of transformed cancer cells to up-regulate autophagy, a cellular stress response [3]. To date, a molecular basis for the control of autophagy by eEF-2K has not been described. We have shown that the systemic down-regulation of eEF-2K induces growth retardation of breast cancer tumors in an orthotopic model and also sensitizes the tumors to doxorubicin treatment. While it still remains unclear precisely how the down-regulation of eEF-2K affects these changes, our data suggest that eEF-2K may promote the expression of certain proteins associated with tumorigenesis in breast cancer cells. A similar mechanism may underlie the observed effects of down-regulating eEF-2K on autophagy. Previously, it was suggested that the activation of eEF-2K increases the expression of CaMK-II [63] and Arc [64] in neuronal cells, potentially by increasing CAP-dependent translation [65]. In this study it is notable that several of the proteins down-regulated upon targeting eEF-2K, including cyclin D1 [66], c-Myc [67] and Bcl-2 [68], contain a highly structured *5′*-untranslated region [69] whose expression may be sensitive to rates of translation. Thus, it is possible that targeting eEF-2K directly inhibits the efficient translation of these and other proteins critical for tumorigenesis and possibly autophagy (Figure 8). However, it should be noted that less direct mechanism are also possible. For example, it has been proposed that the transient inhibition of protein synthesis may result in the induction of expression of proto-oncogenes such as c-Myc due to a decrease in the levels of certain short-lived negative growth regulator repressor proteins [70]. It is also interesting to note that transformed cell lines overexpress eEF2 as well as eEF-2K leading to elevated levels of phosphorylated eEF2 [17], [71], [72]. Based on our understanding of the literature, we cannot exclude the possibility that phosphorylated eEF2 directly up-regulates pathways associated with transformation.

### Conclusion

We have shown for the first time that the targeting of eEF-2K in an *in vivo* orthotopic model inhibits growth of established breast cancer tumors and sensitizes the tumors to doxorubicin, an important agent in a number of chemotherapy regimens. Our data suggest that eEF-2K may enhance tumorigenesis through the up-regulation of pro-tumorigenic proteins and pathways including cyclin D1, c-Myc, c-Src/FAK and Akt (Figure 8) whose dysregulation are associated with a poor prognosis in breast cancer [73]–[75].

## Supporting Information

Figure S1 **Downstream molecular effects in breast cancer cells using different siRNA targeting eEF-2K.** Cells were transiently transfected with eEF-2K siRNA, and cell lysates (72 h) were subjected to Western blot analysis. (**A**) eEF-2K down-regulation decreases eEF2 phosphorylation in MCF-7 cells. (**B–C**) Knockdown of eEF-2K using two different siRNAs decreases expression levels of cyclin D1 in MDA-MB-231 cells (B), and c-Myc in MCF-7 cells (C). (**D–E**) eEF-2K knockdown additionally inhibits the activity of Src and FAK as indicated by reduced p-Src (Tyr-416) and p-FAK (Tyr-397) in BT-20 (D) and p-FAK (Tyr-397) in MDA-MB-435 (E) breast cancer cells.
(PDF)

Figure S2 **Effect of knockdown of eEF-2K on cell proliferation.** Cells were transfected with eEF-2K siRNA, and after 48 h proliferation was detected by an MTS assay. Percentage proliferation of (**A**) MDA-MB-231, (**B**) SK-BR3 and (**C**) T47D breast cancer cell lines after treatment with two different siRNA targeting eEF-2K.
(PDF)

Figure S3 **Effect of eEF-2K knockdown on colony formation in MCF-7 cells.** Knockdown of eEF-2K by siRNA (50 nM) significantly inhibited the number of colonies formed. Cells were transfected every 4 days with control or eEF-2K siRNA. An untreated control was concurrently performed.
(PDF)
