# Supplementary material for: Targeted Silencing of Elongation Factor 2 Kinase Suppresses Growth and Sensitizes Tumors to Doxorubicin in an Orthotopic Model of Breast Cancer
Source: PLoS One. 2012 Jul 20;7(7):e41171. doi: 10.1371/journal.pone.0041171 (PMC3401164; doi:10.1371/journal.pone.0041171)
Supplement: Figure S1 — Downstream molecular effects in breast cancer cells using different siRNA targeting eEF-2K. Cells were transiently transfected with eEF-2K siRNA, and cell lysates (72 h) were subjected to Western blot analysis. (A) eEF-2K down-regulation decreases eEF2 phosphorylation in MCF-7 cells. (B–C) Knockdown of eEF-2K using two different siRNAs decreases expression levels of cyclin D1 in MDA-MB-231 cells (B), and c-Myc in MCF-7 cells (C). (D–E) eEF-2K knockdown additionally inhibits the activity of Src and FAK as indicated by reduced p-Src (Tyr-416) and p-FAK (Tyr-397) in BT-20 (D) and p-FAK (Tyr-397) in MDA-MB-435 (E) breast cancer cells. (PDF) [file pone.0041171.s001.pdf]

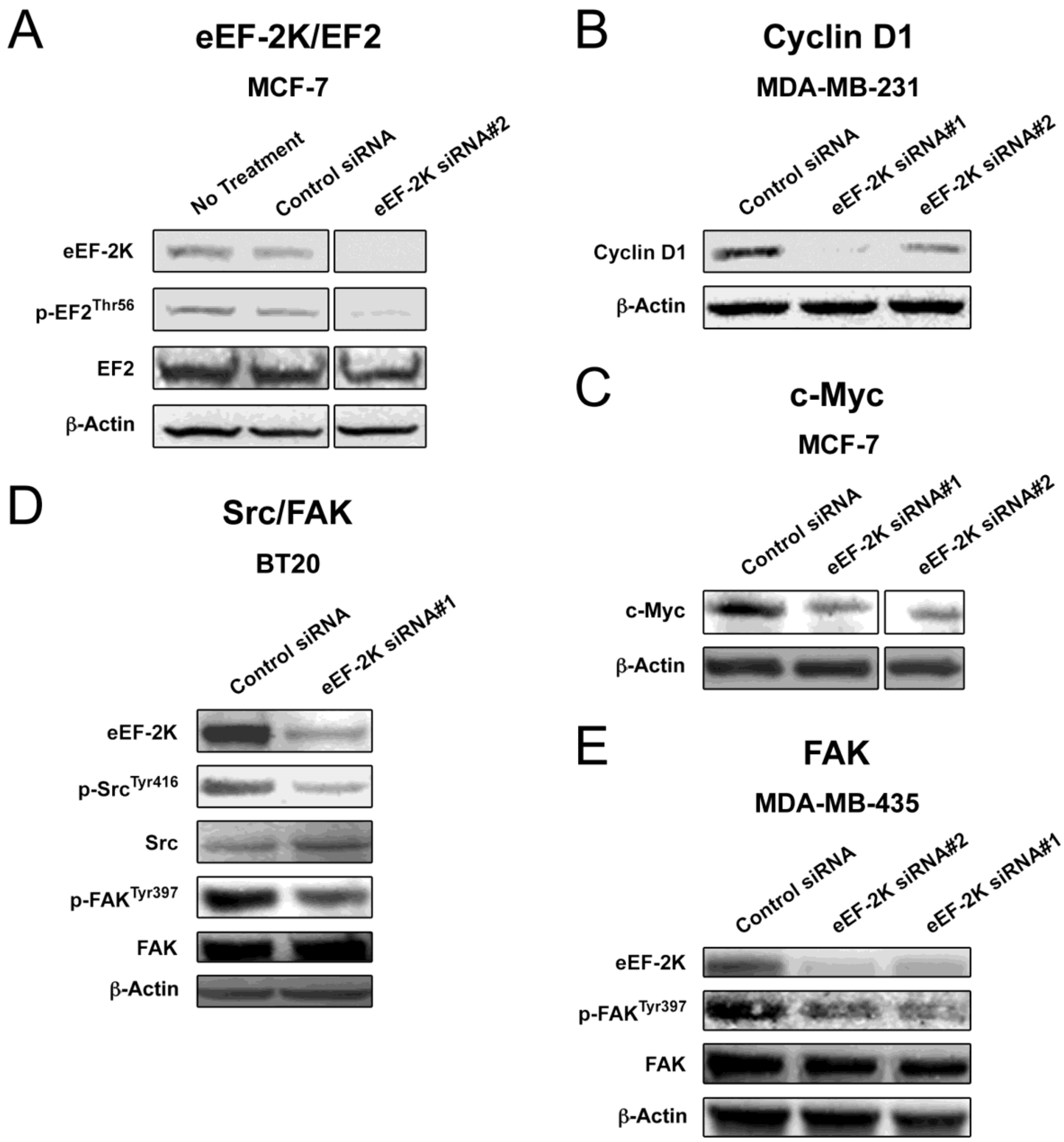

Figure S1. Downstream molecular effects in breast cancer cells using different siRNA targeting eEF-2K. Cells were transiently transfected with eEF-2K siRNA, and cell lysates (72 h) were subjected to Western blot analysis. **(A)** eEF-2K down-regulation decreases eEF2 phosphorylation in MCF-7 cells. **(B-C)** Knockdown of eEF-2K using two different siRNAs decreases expression levels of cyclin D1 in MDA-MB-231 cells (B), and c-Myc in MCF-7 cells (C). **(D-E)** eEF-2K knockdown additionally inhibits the activity of Src and FAK as indicated by reduced p-Src (Tyr-416) and p-FAK (Tyr-397) in BT-20 (D) and p-FAK (Tyr-397) in MDA-MB-435 (E) breast cancer cells.
