# Supplementary material for: Targeted Silencing of Elongation Factor 2 Kinase Suppresses Growth and Sensitizes Tumors to Doxorubicin in an Orthotopic Model of Breast Cancer
Source: PLoS One. 2012 Jul 20;7(7):e41171. doi: 10.1371/journal.pone.0041171 (PMC3401164; doi:10.1371/journal.pone.0041171)
Supplement: Figure S2 — Effect of knockdown of eEF-2K on cell proliferation. Cells were transfected with eEF-2K siRNA, and after 48 h proliferation was detected by an MTS assay. Percentage proliferation of (A) MDA-MB-231, (B) SK-BR3 and (C) T47D breast cancer cell lines after treatment with two different siRNA targeting eEF-2K. (PDF) [file pone.0041171.s002.pdf]

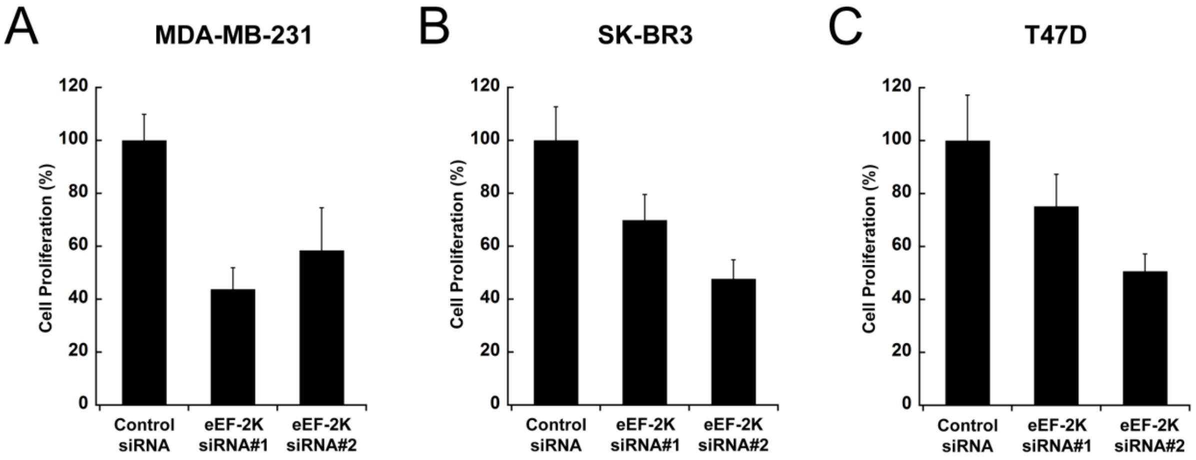

Figure S2. Effect of knockdown of eEF-2K on cell proliferation. Cells were transfected with eEF-2K siRNA, and after 48 h proliferation was detected by an MTS assay. Percentage proliferation of **(A)** MDA-MB-231, **(B)** SK-BR3 and **(C)** T47D breast cancer cell lines after treatment with two different siRNA targeting eEF-2K.
