# Supplementary material for: Targeted Silencing of Elongation Factor 2 Kinase Suppresses Growth and Sensitizes Tumors to Doxorubicin in an Orthotopic Model of Breast Cancer
Source: PLoS One. 2012 Jul 20;7(7):e41171. doi: 10.1371/journal.pone.0041171 (PMC3401164; doi:10.1371/journal.pone.0041171)
Supplement: Figure S3 — Effect of eEF-2K knockdown on colony formation in MCF-7 cells. Knockdown of eEF-2K by siRNA (50 nM) significantly inhibited the number of colonies formed. Cells were transfected every 4 days with control or eEF-2K siRNA. An untreated control was concurrently performed. (PDF) [file pone.0041171.s003.pdf]

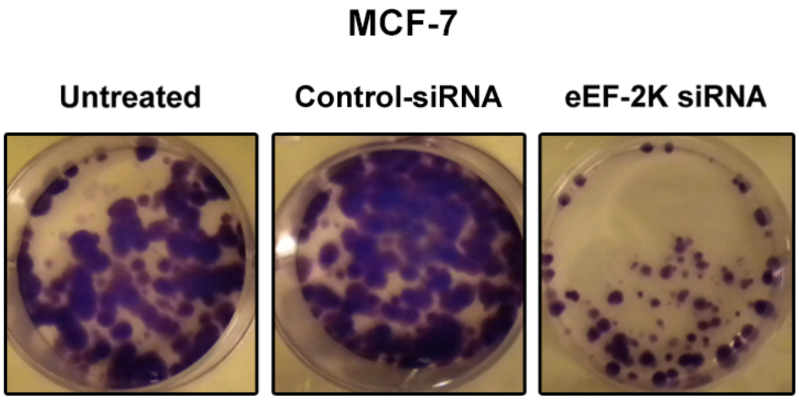

Figure S3. Effect of eEF-2K knockdown on colony formation in MCF-7 cells. Knockdown of eEF-2K by siRNA (50 nM) significantly inhibited the number of colonies formed. Cells were transfected every 4 days with control or eEF-2K siRNA. An untreated control was concurrently performed.
